# Haplotypes of the Ovine Adiponectin Gene and Their Association with Growth and Carcass Traits in New Zealand Romney Lambs

**DOI:** 10.3390/genes8060160

**Published:** 2017-06-12

**Authors:** Qingming An, Huitong Zhou, Jiang Hu, Yuzhu Luo, Jon G. H. Hickford

**Affiliations:** 1 Faculty of Wujiang, Tongren University, Tongren 554300, China; anqingming2009@163.com; 2 Gansu Key Laboratory of Herbivorous Animal Biotechnology, Faculty of Animal Science and Technology, Gansu Agricultural University, Lanzhou 730070, China; Zhou@lincoln.ac.nz (H.Z.); huj@gsau.edu.cn (J.H.); 3 Gene-Marker Laboratory, Faculty of Agriculture and Life Sciences, PO Box 84, Lincoln University, Lincoln 7646, New Zealand

**Keywords:** adiponectin gene (*ADIPOQ*), variation, haplotype, growth traits, carcass traits

## Abstract

Adiponectin plays an important role in energy homeostasis and metabolism in mammalian adipose tissue. In this study, the relationship between adiponectin gene (*ADIPOQ*) haplotypes and variation in growth and carcass traits in New Zealand (NZ) Romney lambs was investigated using General Linear Models (GLMs). Eight haplotypes were found in these lambs and they were composed of the four previously reported promoter fragment sequences (*A_1_*–*D_1_*) and three previously reported intron 2–exon 3 sequences (*A_3_*–*C_3_*). The frequencies of the haplotypes ranged from 0.07% to 45.91%. The presence of *A_1_*–*A_3_* was associated with a decreased pre-weaning growth rate (*p* = 0.037), and decreased leg lean-meat yield (*p* = 0.001), loin lean-meat yield (*p* = 0.018) and total lean-meat yield (*p* = 0.004). The presence of *A_1_*–*C_3_* was associated with increased carcass fat depth over the 12th rib (V-GR; *p* = 0.001) and a decreased proportion of loin lean-meat yield (*p* = 0.045). The presence of *B_1_*–*A_3_* was associated with an increased proportion of leg lean-meat yield (*p* = 0.016) and proportion of shoulder lean-meat yield (*p* = 0.030). No associations were found with birth weight, tailing weight and weaning weight. These results suggest that ovine *ADIPOQ* may have value as a genetic marker for NZ Romney sheep breeding.

## 1. Introduction

Adiponectin (ADIPOQ) is a member of the adipocytokine family of proteins and is secreted primarily by white adipose tissue. It is also produced by other tissues including bone marrow, brown adipose tissue and skeletal muscle [1,2,3]. It has been demonstrated that ADIPOQ plays an important role in regulating energy homeostasis, and in glucose metabolism and lipid metabolism in humans [4,5]. ADIPOQ levels in adipose tissue are negatively correlated with obesity through the modulation of lipid synthesis, glucose utilisation and fatty acid oxidation [6,7]. ADIPOQ may therefore be an important regulator of growth and carcass traits in livestock.

The human adiponectin gene (*ADIPOQ*) was first identified in 1995 and is located on chromosome 3q27 [8,9]. It spans approximately 17 kb and contains three exons (exon 1 and part of exon 2 are non-coding and produce the 5’-untranslated region) and two introns. Several single nucleotide polymorphisms (SNPs) in the human gene have been reported and these SNPs have been associated with obesity, type 2 diabetes susceptibility, cancer risk, serum adiponectin levels and Chronic Obstructive Pulmonary Disease [10,11,12,13].

In livestock species, *ADIPOQ* polymorphisms have been associated with various traits including: measures of adiposity in horses [14], chest circumference in goats [15], fat deposition and various carcass and reproductive traits in pigs [16,17] and meat marbling, ribeye muscle area and carcass fat thickness in cattle [3]. Despite these findings there have been no reports to date of associations with production traits in sheep, although the reports from other common livestock species suggest this may be likely.

Recently, we reported, variation in different regions (spanning the promoter region to the 3’-UTR region) of ovine *ADIPOQ* and described nine separate haplotypes spanning exon 1 to exon 3 [18]. In this study, we used Polymerase Chain Reaction Single-Stranded Conformational Polymorphism (PCR-SSCP) analyses to investigate associations between ovine *ADIPOQ* haplotypes and various production traits in New Zealand (NZ) Romney lambs.

## 2. Materials and Methods

All research involving animals was carried out in accordance with the Animal Welfare Act 1999 (New Zealand Government) and the collection of sheep blood drops by nicking sheep ears is covered by Section 7.5 Animal Identification, of the Animal Welfare (Sheep and Beef Cattle) Code of Welfare2010; a code of welfare issued under the Animal Welfare Act 1999 (New Zealand Government).

### 2.1. Sheep Investigated and Data Collection

In total 1368 NZ Romney lambs from one farm and the progeny of 17 independent sire-lines were investigated. The weights of all the lambs were recorded at birth, tailing (approximately 3 weeks) and weaning (approximately 3 months), and the pre-weaning growth rates were calculated as gained per day (g/day). Gender, sire and birth rank (whether the animal was born single, twin or triplet) were recorded at birth.

In all lambs, male lambs only were slaughtered based on their live weight having exceeded 36 kg for meat production and were used to investigate associations between haplotypes of *ADIPOQ* and growth and carcass muscle traits, whereas both male and female lambs were used to analyse associations with growth traits.

Hot carcass weights (H-W) were measured directly at slaughter. H-W is the weight of the carcass minus the head, pelt and gut. Other carcass muscle traits were subsequently collected using the VIAScan^®^ video imaging analysis system (developed by Meat and Livestock Australia). Carcass fat depth over the 12th rib (V-GR), shoulder lean-meat yield, loin lean-meat yield and leg lean-meat yield were calculated, and represents the percentage of lean tissue as a proportion of the H-W. Total lean-meat yield is the sum of the individual leg, loin and shoulder lean-meat yields for any given carcass. The proportion yield of leg, loin or shoulder is the yield of the specific area divided by the total yield and expressed as a percentage.

Of the 1368 lambs originally born and for which blood was collected, growth and genotype data were subsequently collected for 1185 lambs. Of these lambs, a small number of lambs were excluded from the association analyses. Sample numbers therefore vary slightly in the different analyses undertaken.

### 2.2. Blood Sample and DNA Purification

A blood sample from each lamb was collected onto an FTA card when tailing, and the DNA was purified according to the two-step method described by Zhou et al. [19].

### 2.3. PCR Amplification and Single-Stranded Conformational Polymorhipsm Analysis

**Figure 1:**
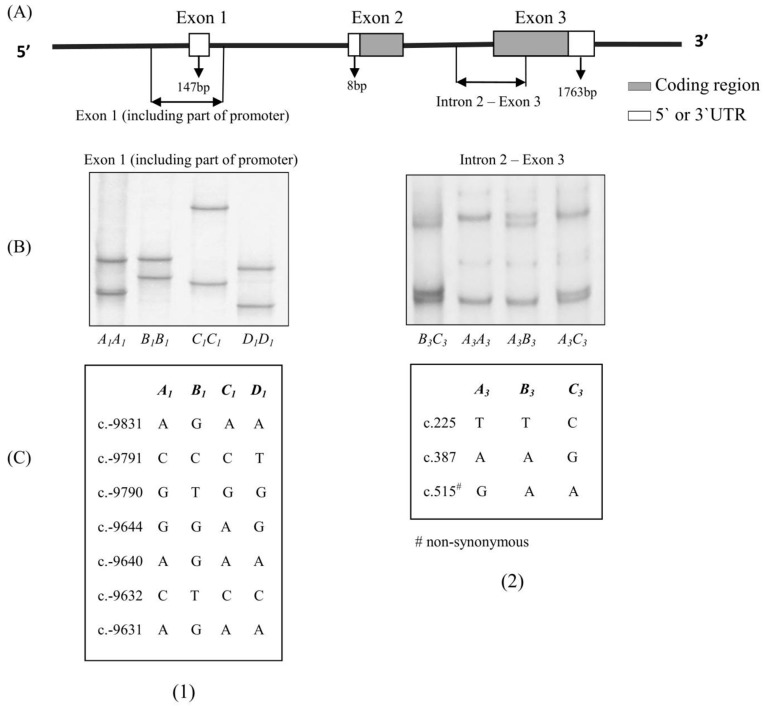
(**A**) Stylistic map showing the regions of the adiponectin gene (*ADIPOQ*) amplified; (**B**) Polymerase Chain Reaction Single-Stranded Conformational Polymorphism (PCR-SSCP) banding patterns for the two regions of ovine *ADIPOQ* amplified; (**C**) Single nucleotide polymorphisms (SNPs) detected for ovine *ADIPOQ* in the two regions. The coordinates of the SNPs are annotated below the patterns based on the ovine whole genome sequence (Gene ID: 101111848) and the numbering of positions follows the guidelines presented at http://www.hgvs.org/mutnomen/.

Two different pairs of primers that have been described previously [18] were used to amplify an exon 1 region and a fragment from the intron 2–exon 3 region of ovine *ADIPOQ* (See Figure 1).

Amplifications were performed in a 15 µL reaction containing the DNA on one punch of FTA card, 150 µM of each deoxynucleotide (dNTP) (Bioline, London, UK), 0.25 µM of each primer, 0.5 U of Taq DNA polymerase (Qiagen, Hilden, Germany), 2.5 mM Mg^2+^, 1× reaction buffer supplied with the enzyme and double-distilled water (ddH_2_O) to make up volume. The thermal profile consisted of 2 min at 94 °C, followed by 35 cycles of 30 s at 94 °C, annealing for 30 s at 58 °C (the primers for the two regions amplified had the same annealing temperature) and 30 s at 72 °C, with a final extension of 5 min at 72 °C.

A 0.7 µL aliquot of each amplicon was mixed with 7 µL of loading dye (98% formamide, 10 mM EDTA, 0.025% xylene-cyanol, 0.025% bromophenol blue). After denaturation at 95 °C for 5 min, samples were cooled rapidly on wet ice and loaded on to 16 cm × 18 cm, 14% acrylamide: bisacrylamide (37.5:1) (Bio-Rad, Hercules, CA, USA) gels for the amplicons from the exon 1 fragment. These were electrophoresed at 300 V and 17 °C for 19 h in 0.5× TBE buffer. The amplicons from the intron 2–exon 3 fragment were analysed using 12% acrylamide: bisacrylamide (37.5:1) (Bio-Rad) gels at 200 V and 20 °C for 19 h in 0.5× TBE buffer.

After electrophoresis, the gels were silver-stained by the method of Byun et al. [20]. The previously reported SSCP patterns for exon 1 region and partial intron 2–exon 3 region were used as standards for determining the region-specific genotypes of the individual lambs.

### 2.4. Haplotype Determination

To ascertain the *ADIPOQ* haplotypes, progeny that typed as homozygous in either of the regions, could have their haplotypes directly inferred based on the co-inheritance of sequences. For example, for an animal presenting with genotype *A_1_A_1_* in exon 1 and genotype *A_3_B_3_* in intron 2–exon 3, the presence of haplotypes *A_1_*–*A_3_* and *A_1_*–*B_3_* could be directly inferred.

If progeny typed as heterozygous in both regions, their haplotypes could be inferred from their sire haplotype and comparison with other progeny from the same sire. For example, for a sire having a diplotype of *A_1_*–*B_3_*/*B_1_*–*C_3_*, approximately half of its offspring will have the haplotype *A_1_*–*B_3_*, while the other half will have *B_1_*–*C_3_*.

### 2.5. Statistical Analyses

All analyses were performed using MINITAB version 16 (State College, PA, USA). General Linear Models (GLMs) were used to assess the effect of the presence or absence (coded as 1 or 0 respectively) of a single haplotype in a sheep’s diplotype on growth traits (including birth weight, weaning weight and growth rate) and the various carcass traits (including V-GR, hind-leg lean-meat yield, loin lean-meat yield, shoulder lean-meat yield, total carcass lean-meat yield and proportion lean-meat yield of hind leg, loin and shoulder).

Birth rank (single, twin and triplet), sire and gender were fitted as fixed factors in the models that analysed birth weight; Rearing rank (single, twin and triplet), sire, age and gender were fitted as fixed factors in the models that analysed tailing weight and weaning weight; Rearing rank (single, twin and triplet), sire and gender were fitted as fixed factors in the models that analysed pre-weaning growth rate; Rearing rank (single, twin and triplet) and age were fitted as fixed factors in the models that analysed carcass traits. Only male lambs were slaughtered, and the sire of each lamb was fitted as a random factor. For these different production traits, either birth rank was fitted as a fixed factor, or birth weight or rearing rank was fitted as a covariate, depending on which had a bigger effect on individual production traits.

In a second series of multi-haplotype GLMs, any haplotypes that had associations in the single-haplotype models with a *p* value of less than 0.2 (*p* < 0.20) and which could thus potentially impact on the trait, were factored into the models, such that we could determine the independent haplotype effects.

For diplotypes with a frequency greater than 5%, and thus that had an adequate sample size per group, a third series of GLMs (Fixed effects: diplotype, gender and birth rank; Random effect: sire) and multiple pair-wise comparisons between diplotype were performed using Least Significant Difference tests to ascertain the effect of diplotype on growth rate and carcass traits, and with use of a Bonferroni correction for the multiple comparisons being undertaken, to limit the possibility of identifying false positive results.

Unless other indicated, all *p* values were considered statistically when *p* < 0.05 and trends were noted when 0.05 < *p* < 0.20.

## 3. Results

### 3.1. Frequencies of the Ovine ADIPOQ Haplotypes

In the 1185 lambs investigated, eight *ADIPOQ* haplotypes were found. These were composed of the four previously reported exon 1 variants (*A_1_*, *B_1_*, *C_1_* and *D_1_*; GenBank accession numbers: KP903754–KP903757) and three previously reported intron 2–exon 3 variants (*A_3_*, *B_3_* and *C_3_*; GenBank accession numbers: KP903762–KP903764). The PCR-SSCP banding patterns observed for the two fragments are illustrated in Figure 1. The haplotypes were found with the following frequencies: *A_1_*–*A_3_* (36.23%), *A_1_*–*B_3_* (0.53%), *A_1_*–*C_3_* (10.16%), *B_1_*–*A_3_* (45.91%), *B_1_*–*B_3_* (1.47%), *B_1_*–*C_3_* (5.56%), *D_1_*–*A_3_* (0.07%) and *D_1_*–*C_3_* (0.07%).

### 3.2. Association of the Ovine ADIPOQ Haplotypes with Growth Traits

In the single-haplotype (presence/absence) models, the presence of haplotype *A_1_*–*A_3_* was associated with a decreased pre-weaning growth rate (*p* = 0.031). No associations were found between the other haplotypes and either birth weight, tailing weight or weaning weight in the lambs studied (Table 1).

### 3.3. Association of the Ovine ADIPOQ Haplotypes with Carcass Traits

In the single-haplotype (presence/absence) model, the presence of haplotype *A_1_–A_3_* was associated with decreased leg lean-meat yield (*p* = 0.001), loin lean-meat yield (*p* = 0.028), total lean-meat yield (*p* = 0.004) and proportion shoulder lean-meat yield (*p* = 0.011) (Table 2). The presence of haplotype *A_1_–C_3_* was associated with increased V-GR (*p* = 0.001) and proportion of loin lean-meat yield (*p* = 0.048) (Table 2). The presence of haplotype *B_1_–A_3_* was associated with an increased proportion of leg lean-meat yield (*p* = 0.006) and proportion of shoulder lean-meat yield (*p* = 0.037) (Table 2). These haplotype associations remained significant when the other haplotypes where *p* < 0.2 were factored into the models. No associations with the presence (or absence) of *B_1_–C_3_* were detected.

### 3.4. Diplotype Analyses

The diplotypes *A_1_–A_3_*/*A_1_–A_3_* (*n* = 61), *A_1_–A_3_*/*A_1_–C_3_* (*n* = 38), *A_1_–A_3_*/*B_1_–A_3_* (*n* = 140), *B_1_–A_3_*/*B_1_–A_3_* (*n* = 89) and *B_1_–A_3_*/*B_1_–C_3_* (*n* = 24) occurred at a frequency of greater than 5%. *ADIPOQ* diplotype was found to have an overall effect on leg lean-meat yield (*p* = 0.017), loin lean-meat yield (*p* = 0.028) and total lean-meat yield (*p* = 0.037) (Table 3). The *A_1_–A_3_*/*A_1_–A_3_* diplotype had a lower mean leg, loin and total lean-meat yield than those with the diplotypes *A_1_–A_3_*/*A_1_–C_3_*, *A_1_–A_3_*/*B_1_–A_3_*, *B_1_–B_3_*/*B_1_–B_3_* and *B_1_–A_3_*/*B_1_–C_3_* (Table 3). Diplotype was not found to have significant effect on V-GR or proportion of loin and shoulder lean-meat yield, but a trend was evident (*p* = 0.062, *p* = 0.094 and *p* = 0.061, respectively) with the *A_1_–A_3_*/*A_1_–C_3_* diplotype having a higher mean V-GR and proportion of loin lean-meat yield, than the other diplotypes (Table 3).

No association was found between the *ADIPOQ* diplotype and mean birth weight, tailing weight, weaning weight and pre-weaning growth rate (results not shown).

## 4. Discussion

This is the first study to report associations between ovine *ADIPOQ* haplotypes and selected growth and carcass traits in lambs and the findings suggest that this genetic variation may play a role in traits that are of economic importance to sheep farmers.

Eight haplotypes were detected, which is less than reported previously [18], with haplotype *C_1_*–*C_3_* being described in Merino sheep [18], but not being found in the NZ Romney lambs in this study. This may simply be because of the origin of the sheep selected for investigation, or alternatively because *C_1_*–*C_3_* has been selected against in the NZ Romney sheep population. Clarifying this apparent breed difference will require further study of *ADIPOQ* in both more NZ Romney and Merino sheep, and other breeds as well. Given the variation reported to date and the limited number of sheep investigated here and by An et al. [18], it might be anticipated that further variation in ovine *ADIPOQ* may be found.

In lamb production systems, pre-weaning growth rate is considered to be a critically important factor in determining the efficiency of the system, as faster growing lambs have a proportionally reduced maintenance cost if they reach slaughter weight more rapidly. In this study, the results suggest that if lambs are weaned at 100 days of age, in the absence of a birth weight effect, the presence of haplotype *A_1_*–*A_3_* is associated with a reduction in growth rate of approximately 7.7 grams per day (275.9–268.2 g/day), or 770 grams from birth to weaning (estimated from Table 1). This suggests that selection away from the *A_1_*–*A_3_* haplotype could be of benefit to sheep breeding, especially as this haplotype also does not seem to benefit carcass composition traits.

In this study, the absence of *A_1_*–*A_3_* also was associated with an increase in leg, loin and total lean-meat yield of 0.45 ± 0.22%, 0.08 ± 0.06% and 0.71 ± 0.14%, respectively (estimated from Table 2). Thus, for an 18 kg lamb carcass, these yield improvements would equate to an additional 128 g of lean-meat (estimated from Table 2). Although this may seem small, it may have a commercial benefit because of its location in the carcass. The loin is typically considered a high value part of a carcass, as it is part of valuable carcass cuts, including the Frenched-rack of lamb and bone-out “backstrap”.

The presence of haplotype *A_1_*–*C_3_* was associated with increased V-GR, or increased fat depth (absent: 2.87 ± 0.33 mm;present: 4.24 ± 0.42 mm; see Table 2). Excessive carcass fat cover is undesirable in some markets, especially in the context of high fat intake diets being associated with obesity, diabetes and heart disease [21]. Accordingly breeding away from haplotype *A_1_*–*C_3_* might reduce carcass cover and provide a benefit in markets where lean meat is desirable.

A haplotype is a particular combination of alleles or variant sequences. Their value in genome analysis is that they help increase the Polymorphism Information Content (PIC) [22] and they are also considered to improve the power of genome-wide association studies [23]. In this study, the SNPs described in these variants of the fragment spanning exon 1 (including part of promoter) of *ADIPOQ* (c.-9831A/G, c.-9791C/T, c.-9790G/T, c.-9644G/A, c.-9640A/G, c.-9632C/T and c.-9631A/G) were all located in the promoter region and the SNPs described in these variants of intron 2–exon 3 of *ADIPOQ* (c.225T/C, c.387A/G and c.515G/A) were all located in the coding region (See Figure 1).

The promoter of *ADIPOQ* includes physiologically important binding site for stimulatory protein 1 (Sp1), sterol-regulatory element binding protein (SREBP), activator protein 1 (AP1) and CCAAT-enhancer binding protein (C/EBP) [24], and in humans a substitution (c.-11377C/G) has been reported. This substitution is located in a SP1 binding site and it results in a change in SP1 bonding and causes a reduction in ADIPOQ activity [25]. Three other substitutions in the promoter (c.-19166T/G, c.-11426A/G and c.-11391G/A) have also been detected in humans and reported to be associated with type 2 diabetes and obesity [26].

Shin et al. [27] reported that the SNP c.-10073A/G in the bovine *ADIPOQ* promoter is associated with *longissimus dorsi* muscle area and back-fat thickness in Hanwoo (Korean) cattle, and c.-10096C/T and c.-9931G/A in the bovine *ADIPOQ* promoter have been associated with fat thickness and ribeye muscle area in Angus cattle [3]. In pigs, the SNPs c.-9002G/A and c.-9767C/T in the promoter region segregate as two haplotypes (G-C and A-T) and these have been associated with loin muscle depth and loin eye area [28].

One SNP reported here (c.515G/A) was located in the exon 3 coding region and result in the amino acid changes p.Lys172Arg. This is in a globular domain of ovine ADIPOQ. The substitution (rs17366743 C/T) in human *ADIPOQ* exon 3, which is proximal to ovine c.515G/A, has been reported to be associated with obesity and variation in adiponectin levels in some populations [29]. This would support the contention that c.515G/A may associated with carcass fat traits in sheep.

This evidence from above and the results of exon 1 and intron 2–exon 3 fragment support the contention that the variation described here may affect key sheep traits, such as potentially fat deposition, fat composition and meat quality. So, at the same time indicated that haplotype is more comprehensive and reliable than genotype or allele in genome-wide association studies.

In this study, the diplotypes were associated with various carcass traits (*p* < 0.05; see Table 3). It is noteworthy that the homozygous and heterozygous diplotypes containing *A_1_*–*A_3_* have lower leg, loin and total lean-meat yield than in the absence of *A_1_*–*A_3_*. This is consistent with the individual haplotype presence/absence models. The diplotype containing *A_1_*–*C_3_* is associated with a higher V-GR than other diplotypes, and this too is consisted with the earlier presence/absence models. Overall the results suggest that the presence of haplotype *A_1_*–*A_3_* and the absence of *A_1_*–*C_3_* are important factors in selecting better yielding, but leaner lambs.

Overall, it could be concluded that further investigation of *ADIPOQ* variation and haplotypes in different sheep breeds is worthwhile, especially as only one breed and small number of lambs were investigated in this study. The results reported here are consistent with prior observations in cattle and pigs, and together they would suggest that *ADIPOQ* variation could be used for marker-assisted selection for improved carcass traits in sheep. Further study is required to confirm these preliminary finding.

## Figures and Tables

**Table 1:** Association of ovine adiponectin gene (*ADIPOQ*) haplotypes with growth traits in NZ Romney sheep.

Traits (unit)	Haplotype Assessed	Mean ± SE ^a^				*p*-Value
		Haplotype Absent (0)	*n*	Haplotype Present (1)	*n*	
Birth weight (kg)	*A_1_–A_3_*	5.28 ± 0.15	384	5.30 ± 0.15	561	0.721
	*A_1_–C_3_*	5.31 ± 0.15	769	5.22 ± 0.16	176	0.248
	*B_1_–A_3_*	5.24 ± 0.15	284	5.32 ± 0.15	661	0.203
	*B*_1_–*C*_3_	5.30 ± 0.15	873	5.20 ± 0.17	72	0.264
Tailing weight (kg)	*A_1_–A_3_*	12.22 ± 0.25	384	12.13 ± 0.24	561	0.586
	*A*_1_–*C*_3_	12.19 ± 0.23	769	12.02 ± 0.29	176	0.436
	*B*_1_–*A*_3_	12.15 ± 0.26	284	12.17 ± 0.23	661	0.885
	*B*_1_–*C*_3_	12.14 ± 0.23	873	12.57 ± 0.37	72	0.293
Weaning weight (kg)	*A_1_–A_3_*	27.91 ± 0.41	384	27.46 ± 0.39	561	0.161
	*A*_1_–*C*_3_	27.63 ± 0.38	769	27.69 ± 0.49	176	0.884
	*B*_1_–*A*_3_	27.58 ± 0.43	284	27.68 ± 0.39	661	0.745
	*B*_1_–*C*_3_	27.62 ± 0.38	873	28.05 ± 0.61	72	0.405
Pre-weaning growth rate (g/day)	***A*_1_–*A*_3_**	**275.9 ± 8.54**	**384**	**268.2 ± 8.56**	**561**	**0.025**
	*A*_1_–*C*_3_	272.6 ± 8.46	769	270.5 ± 9.32	176	0.684
	*B*_1_–*A*_3_	271.7 ± 8.73	284	272.4 ± 8.49	661	0.844
	*B*_1_–*C*_3_	272.0 ± 8.41	873	274.8 ± 9.97	72	0.633

^a^ Estimated marginal means and standard errors (SE) derived from General Linear Models (GLMs) with haplotype presence/absence, gender and birth rank fitted as fixed factors and sire group fitted as a random factor *p* < 0.05 in bold). NZ: New Zealand.

**Table 2:** Association of ovine *ADIPOQ* haplotypes with various carcass traits in NZ Romney sheep.

Traits (unit)	Haplotype Assessed	Other Haplotype in Model	Mean ± SE ^a^				*p*-Value
			Haplotype Absent (0)	*n*	Haplotype Present (1)	*n*	
V-GR (mm) ^b^	*A*_1_–*A*_3_	*—*	3.18 ± 0.37	159	3.10 ± 0.35	244	0.771
	***A*_1_–*C*_3_**	*—*	**2.87 ± 0.33**	**330**	**4.24 ± 0.42**	**73**	**0.001**
	*B_1_–A_3_*	*—*	3.39 ± 0.37	127	2.98 ± 0.35	276	0.136
	*B*_1_–*C*_3_	*—*	2.26 ± 0.47	369	2.56 ± 0.64	34	0.738
	***A*_1_–*C*_3_**	***B_1_–A_3_***	**2.92 ± 0.39**	**330**	**4.43 ± 0.49**	**73**	**0.001**
	*B_1_–A_3_*	*A_1_**–**C_3_*	3.56 ± 0.42	127	3.79 ± 0.45	276	0.478
Leg lean-meat yield (%) ^c^	***A*_1_–*A*_3_**	*—*	**20.75 ± 0.16**	**159**	**20.30 ± 0.15**	**244**	**0.001**
	*A*_1_–*C*_3_	*—*	20.50 ± 0.15	330	20.39 ± 0.19	73	0.450
	*B_1_–A_3_*	*—*	20.30 ± 0.17	127	20.59 ± 0.16	276	0.023
	*B*_1_–*C*_3_	*—*	20.97 ± 0.21	369	20.85 ± 0.29	34	0.585
	***A*_1_–*A*_3_**	***B_1_–A_3_***	**20.65 ± 0.18**	**159**	**20.18 ± 0.19**	**244**	**0.001**
	*B_1_–A_3_*	*A_1_**–**A_3_*	20.36 ± 0.19	127	20.47 ± 0.20	276	0.467
Loin lean-meat yield (%) ^c^	***A*_1_–*A*_3_**	*—*	**14.12 ± 0.11**	**159**	**13.94 ± 0.11**	**244**	**0.028**
	*A*_1_–*C*_3_	*—*	14.01 ± 0.11	330	14.04 ± 0.13	73	0.723
	*B*_1_–*A*_3_	*—*	13.98 ± 0.12	127	14.03 ± 0.11	276	0.598
	*B*_1_–*C*_3_	*—*	14.28 ± 0.15	369	14.34 ± 0.21	34	0.565
Shoulder lean-meat yield (%) ^c^	*A_1_–A_3_*	*—*	16.54 ± 0.13	159	16.46 ± 0.13	244	0.399
	*A*_1_–*C*_3_	*—*	16.52 ± 0.12	330	16.36 ± 0.15	73	0.183
	*B*_1_–*A*_3_	*—*	16.51 ± 0.14	127	16.48 ± 0.13	276	0.786
	*B*_1_–*C*_3_	*—*	17.01 ± 0.17	369	17.07 ± 0.23	34	0.808
Total lean-meat yield (%) ^d^	***A*_1_–*A*_3_**	*—*	**51.42 ± 0.34**	**159**	**50.71 ± 0.32**	**244**	**0.004**
	*A*_1_–*C*_3_	*—*	51.03 ± 0.32	330	50.79 ± 0.40	73	0.448
	*B*_1_–*A*_3_	*—*	50.80 ± 0.35	127	51.10 ± 0.33	276	0.245
	*B*_1_–*C*_3_	*—*	52.16 ± 0.44	369	52.26 ± 0.59	34	0.583
Proportion leg lean-meat yield (%) ^e^	***A_1_–A_3_***	***—***	**40.36 ± 0.16**	**159**	**40.04 ± 0.14**	**244**	**0.004**
	*A*_1_–*C*_3_	*—*	40.17 ± 0.15	330	40.14 ± 0.18	73	0.806
	***B*_1_–*A*_3_**	*—*	**39.96 ± 0.16**	**127**	**40.29 ± 0.15**	**276**	**0.006**
	*B*_1_–*C*_3_	*—*	40.00 ± 0.21	369	39.89 ± 0.29	34	0.838
	***A_1_–A_3_***	***B_1_–A_3_***	**40.27 ± 0.17**	**159**	**40.00 ± 0.19**	**244**	**0.038**
	***B_1_–A_3_***	***A_1_–A_3_***	**39.99 ± 0.18**	**127**	**40.28 ± 0.19**	**276**	**0.046**
Proportion loin lean-meat yield (%) ^e^	*A*_1_–*A*_3_	*—*	27.47 ± 0.13	159	27.50 ± 0.12	244	0.746
	***A_1_–C_3_***	***—***	**27.45 ± 0.12**	**330**	**27.75 ± 0.15**	**73**	**0.048**
	*B*_1_–*A*_3_	*—*	27.53 ± 0.13	127	27.46 ± 0.12	276	0.458
	*B*_1_–*C*_3_	*—*	27.37 ± 0.17	369	27.43 ± 0.23	34	0.836
Proportion shoulder lean-meat yield (%) ^e^	***A_1_–A_3_***	**—**	**32.17 ± 0.16**	**159**	**32.46 ± 0.15**	**244**	**0.011**
	*A*_1_–*C*_3_	*—*	32.38 ± 0.15	330	32.21 ± 0.19	73	0.257
	***B*_1_–*A*_3_**	*—*	**32.51 ± 0.16**	**127**	**32.25 ± 0.15**	**276**	**0.037**
	*B*_1_–*C*_3_	*—*	32.63 ± 0.22	369	32.68 ± 0.29	34	0.713
	*A_1_–A_3_*	*B_1_**–**A_3_*	32.18 ± 0.18	159	32.37 ± 0.19	244	0.147
	***B*_1_–*A*_3_**	***A_1_–A_3_***	**32.45 ± 0.18**	**127**	**32.11 ± 0.19**	**276**	**0.024**

^a^ Estimated marginal means and standard errors (SE) derived from General Linear Models (GLMs) with haplotype presence/absence, gender and birth rank fitted as fixed factors and sire group fitted as a random factor (*p* < 0.05 in bold). ^b^ V-GR represents carcass fat depth over the 12th rib. ^c^ Lean-meat yield expressed as a percentage of hot carcass weight. ^d^ Total lean-meat yield is the sum of the leg, loin and shoulder lean-meat yield. ^e^ The proportion lean-meat yield of leg, loin or shoulder lean-meat is the yield of the specific area divided by the total yield expressed as a percentage.

**Table 3:** Association of ovine *ADIPOQ* diplotypes with various carcass traits in NZ Romney sheep.

Traits (unit)	Mean ± SE ^a^					*p-*Value
	*A_1_–A_3_*/*A_1_–A_3_* (*n* = 61)	*A_1_–A_3_*/*A_1_–C_3_* (*n* = 38)	*A_1_–A_3_*/*B_1_–A_3_* (*n* = 140)	*B_1_–A_3_*/*B_1_–A_3_* (*n* = 89)	*B_1_–A_3_*/*B_1_–C_3_* (*n* = 24)	
V-GR (mm) ^b^	1.65 ± 0.68 ^b^	3.22 ± 0.74 ^a^	2.01 ± 0.64 ^a,b^	1.94 ± 0.63 ^a,b^	2.25 ± 0.77 ^a,b^	*0.062*
Leg lean-meat yield (%) ^c^	**20.80 ± 0.31 ^b^**	**20.86 ± 0.34 ^a,b^**	**20.96 ± 0.29 ^b^**	**21.41 ± 0.29 ^a^**	**21.11 ± 0.35 ^a,b^**	**0.017**
Loin lean-meat yield (%) ^c^	**14.19 ± 0.22 ^b^**	**14.54 ± 0.25 ^a,b^**	**14.30 ± 0.21 ^a,b^**	**14.59 ± 0.21 ^a^**	**14.40 ± 0.26 ^a,b^**	**0.028**
Shoulder lean-meat yield (%) ^c^	17.21 ± 0.25	17.07 ± 0.28	17.03 ± 0.24	17.25 ± 0.23	17.22 ± 0.29	0.347
Total lean-meat yield (%) ^d^	**52.20 ± 0.65 ^a,b^**	**52.47 ± 0.71 ^a,b^**	**52.29 ± 0.61 ^b^**	**53.24 ± 0.60 ^a^**	**52.73 ± 0.74 ^a,b^**	**0.037**
Proportion leg lean-meat yield (%) ^e^	39.82 ± 0.31	39.73 ± 0.34	40.07 ± 0.29	40.19 ± 0.28	40.02 ± 0.35	0.227
Proportion loin lean-meat yield (%) ^e^	27.18 ± 0.25 ^b^	27.73 ± 0.27 ^a^	27.35 ± 0.23 ^ab^	27.39 ± 0.23 ^a,b^	27.31 ± 0.28 ^a,b^	*0.094*
Proportion shoulder lean-meat yield (%) ^e^	33.00 ± 0.32 ^a^	32.54 ± 0.35 ^a,b^	32.58 ± 0.30 ^a,b^	32.42 ± 0.29 ^b^	32.67 ± 0.36 ^a,b^	*0.061*

^a^ Estimated marginal means and standard errors (SE) derived from General Linear Models (GLMs) with haplotype presence/absence, gender and birth rank fitted as fixed factors and sire group fitted as a random factor. Means within a row that do not share a superscript letter are different at *p* < 0.05 (*p* < 0.05 in bold and 0.05 ≤ *p* < 0.10 in italics). ^b^ V-GR represents carcass fat depth over the 12th rib. ^c^ Lean-meat yield expressed as a percentage of hot carcass weight. ^d^ Total lean-meat yield is the sum of the leg, loin and shoulder lean-meat yield. ^e^ The proportion lean-meat yield of leg, loin or shoulder lean-meat is the yield of the specific area divided by the total yield expressed as a percentage.

## References

[1] Shinoda Y. (2006). Regulation of bone formation by adiponectin through autocrine/paracrine and endocrine pathways. Nippon Rinsho Jpn. J. Clin. Med..

[2] Kadowaki T., Yamauchi T. (2005). Adiponectin and adiponectin receptors. Endocr. Rev..

[3] Morsci N.S., Schnabel R.D., Taylor J.F. (2006). Association analysis of adiponectin and somatostatin polymorphisms on BTA1 with growth and carcass traits in Angus cattle. Anim. Genet..

[4] Berg A.H., Combs T.P., Du X., Brownlee M., Scherer P.E. (2001). The adipocyte-secreted protein Acrp30 enhances hepatic insulin action. Nat. Med..

[5] Yamauchi T., Kamon J., Waki H., Terauchi Y., Kubota N., Hara K., Mori Y., Ide T., Murakami K., Tsuboyama-Kasaoka N. (2001). The fat-derived hormone adiponectin reverses insulin resistance associated with both lipoatrophy and obesity. Nat. Med..

[6] Arita Y. (2000). Genomic structure and mutations in adipose-specific gene, adiponectin. Int. J. Obes..

[7] Dall’Olio S., Davoli R., Buttazzoni L., Zambonelli P., Russo V. (2009). Study of porcine adiponectin (ADIPOQ) gene and association of a missense mutation with EBVs for production and carcass traits in Italian Duroc heavy pigs. Livest. Sci..

[8] Scherer P.E., Williams S., Fogliano M., Baldini G., Lodish H.F. (1995). A Novel Serum Protein Similar to C1q, Produced Exclusively in Adipocytes. J. Biol. Chem..

[9] Hsueh W.C., St Jean P.L., Mitchell B.D., Pollin T.I., Knowler W.C., Ehm M.G., Bell C.J., Sakul H., Wagner M.J., Burns D.K. (2003). Genome-wide and fine-mapping linkage studies of type 2 diabetes and glucose traits in the Old Order Amish: Evidence for a new diabetes locus on chromosome 14q11 and confirmation of a locus on chromosome 1q21-q24. Diabetes.

[10] Chu H., Wang M., Zhong D., Shi D., Ma L., Tong N., Zhang Z. (2013). AdipoQ polymorphisms are associated with type 2 diabetes mellitus: A meta-analysis study. Diabetes.

[11] Yang Y., Zhang F., Ding R., Skrip L., Wang Y., Lei H., Hu D. (2013). ADIPOQ gene polymorphisms and cancer risk: A meta-analysis. Cytokine.

[12] Ramya K., Ayyappa K.A., Ghosh S., Mohan V., Radha V. (2013). Genetic association of ADIPOQ gene variants with type 2 diabetes, obesity and serum adiponectin levels in south Indian population. Gene.

[13] Yuan Y., Jiang H., Kuang J., Hou X., Feng Y., Su Z. (2012). Genetic variations in ADIPOQ gene are associated with chronic obstructive pulmonary disease. PLoS ONE.

[14] Kearns C.F., Mckeever K.H., Roegner V., Brady S.M., Malinowski K. (2006). Adiponectin and leptin are related to fat mass in horses. Vet. J..

[15] Fang X., Du Y., Zhang C., Shi X., Chen D., Sun J., Jin Q., Lan X., Chen H. (2011). Polymorphism in a microsatellite of the acrp30 gene and its association with growth traits in goats. Biochem. Genet..

[16] Dai L.H., Xiong Y.Z., Deng C.Y., Jiang S.W., Zuo B., Zheng R., Li F.E., Lei M.G. (2006). Association of the A-G polymorphism in porcine adiponectin gene with fat deposition and carcass traits. Asian-Aust. J. Anim. Sci..

[17] Houde A.A., Murphy B.D., Mathieu O., Bordignon V., Palin M.F. (2008). Characterization of swine adiponectin and adiponectin receptor polymorphisms and their association with reproductive traits. Anim. Genet..

[18] An Q.M., Zhou H.T., Hu J., Luo Y.Z., Hickford J.G. (2015). Haplotypes and Sequence Variation in the Ovine Adiponectin Gene (ADIPOQ). Genes.

[19] Zhou H.T., Hickford J.G., Fang Q. (2006). A two-step procedure for extracting genomic DNA from dried blood spots on filter paper for polymerase chain reaction amplification. Anal. Biochem..

[20] Byun S.O., Fang Q., Zhou H.T., Hickford J.G. (2009). An effective method for silver-staining DNA in large numbers of polyacrylamide gels. Anal. Biochem..

[21] Volk M.G. (2007). An examination of the evidence supporting the association of dietary cholesterol and saturated fats with serum cholesterol and development of coronary heart disease. Altern. Med. Rev..

[22] Gibbs R.A., Belmont J.W., Hardenbol P., Willis T.D., Yu F., Yang H., Chang L.Y., Huang W., Liu B., Shen Y. (2003). The International HapMap Project. Nature.

[23] Snyder M.W., Adey A., Kitzman J.O., Shendure J. (2015). Haplotype-resolved genome sequencing: Experimental methods and applications. Nat. Rev. Genet..

[24] Barth N., Langmann T., Schölmerich J., Schmitz G., Schäffler A. (2004). Identification of regulatory elements in the human adipose most abundant gene transcript-1 (apM-1) promoter: Role of SP1/SP3 and TNF-α as regulatory pathways. Diabetologia.

[25] Menzaghi C., Ercolino T., Di P.R., Berg A.H., Warram J.H., Scherer P.E., Trischitta V., Doria A. (2002). A haplotype at the adiponectin locus is associated with obesity and other features of the insulin resistance syndrome. Diabetes.

[26] Chung H.F., Long K.Z., Hsu C.C., Mamun A.A., Chiu Y.F., Tu H.P., Chen P.S., Jhang H.R., Hwang S.J., Huang M.C. (2014). Adiponectin gene (ADIPOQ) polymorphisms correlate with the progression of nephropathy in Taiwanese male patients with type 2 diabetes. Diabetes Res. Clin. Pract..

[27] Shin S., Chung E. (2013). Novel SNPs in the bovine ADIPOQ and PPARGC1A genes are associated with carcass traits in Hanwoo (Korean cattle). Mol. Biol. Rep..

[28] Cieslak J., Flisikowska T., Schnieke A., Kind A., Szydlowski M., Switonski M., Flisikowski K. (2012). Polymorphisms in the promoter region of the adiponectin (ADIPOQ) gene are presumably associated with transcription level and carcass traits in pigs. Anim. Genet..

[29] Owecki M., Miczke A., Kaczmarek M., Hoppe-Gołebiewska J., Pupek-Musialik D., Słomski R., Bryll W., Cymerys M., Nikisch E., Sowiński J. (2007). The Y111 H (T415C) polymorphism in exon 3 of the gene encoding adiponectin is uncommon in Polish obese patients. Horm. Metab. Res..

